# Neural responses to reward anticipation and feedback in adult and adolescent cannabis users and controls

**DOI:** 10.1038/s41386-022-01316-2

**Published:** 2022-04-06

**Authors:** Martine Skumlien, Claire Mokrysz, Tom P. Freeman, Matthew B. Wall, Michael Bloomfield, Rachel Lees, Anna Borissova, Kat Petrilli, James Carson, Tiernan Coughlan, Shelan Ofori, Christelle Langley, Barbara J. Sahakian, H. Valerie Curran, Will Lawn

**Affiliations:** 1 Department of Psychiatry, University of Cambridge, Cambridge, UK; 2 Clinical Psychopharmacology Unit, Clinical, Educational and Health Psychology Department, University College London, London, UK; 3 Addiction and Mental Health Group (AIM), Department of Psychology, University of Bath, Bath, UK; 4 Invicro, London, UK; 5 Faculty of Medicine, Department of Metabolism, Digestion and Reproduction, Imperial College London, London, UK; 6 Division of Psychiatry, University College London, London, UK; 7 Behavioural and Clinical Neuroscience Institute, University of Cambridge, Cambridge, UK; 8 National Addiction Centre, Institute of Psychiatry, Psychology & Neuroscience, King’s College London, London, UK

**Keywords:** Human behaviour, Cognitive neuroscience, Reward

## Abstract

Chronic use of drugs may alter the brain’s reward system, though the extant literature concerning long-term cannabis use and neural correlates of reward processing has shown mixed results. Adolescents may be more vulnerable to the adverse effects of cannabis than adults; however, this has not been investigated for reward processing. As part of the ‘CannTeen’ study, in the largest functional magnetic resonance imaging study of reward processing and cannabis use to date, we investigated reward anticipation and feedback in 125 adult (26–29 years) and adolescent (16–17 years) cannabis users (1–7 days/week cannabis use) and gender- and age-matched controls, using the Monetary Incentive Delay task. Blood-oxygen-level-dependent responses were examined using region of interest (ROI) analyses in the bilateral ventral striatum for reward anticipation and right ventral striatum and left ventromedial prefrontal cortex for feedback, and exploratory whole-brain analyses. Results showed no User-Group or User-Group × Age-Group effects during reward anticipation or feedback in pre-defined ROIs. These null findings were supported by post hoc Bayesian analyses. However, in the whole-brain analysis, cannabis users had greater feedback activity in the prefrontal and inferior parietal cortex compared to controls. In conclusion, cannabis users and controls had similar neural responses during reward anticipation and in hypothesised reward-related regions during reward feedback. The whole-brain analysis revealed tentative evidence of greater fronto-parietal activity in cannabis users during feedback. Adolescents showed no increased vulnerability compared with adults. Overall, reward anticipation and feedback processing appear spared in adolescent and adult cannabis users, but future longitudinal studies are needed to corroborate this.

## Introduction

Cannabis is the third most commonly used controlled substance worldwide, after alcohol and nicotine [1]. User rates are disproportionately high among adolescents and young adults [1], with annual prevalence rates estimated at 19.3% among 15-year olds in England [2], and 28.0% of 15–16-year olds in the United States [3]. As cannabis regulation is currently changing in many countries, it is becoming increasingly important to understand if and how long-term cannabis use impacts the brain and cognition, so that new policies can be designed to minimise harm and maximise benefit.

Adolescents may respond differently to cannabis exposure compared to adults. Adolescence is an important period of neurodevelopment, during which neural connections are selectively pruned and strengthened in an experience-driven manner [4, 5]. The endocannabinoid system, the primary neurobiological target of cannabis, is thought to play a central role in this development, and is itself an important target of neuromaturation during adolescence [6]. Therefore, many researchers have suggested that adolescence is a sensitive period for the potentially adverse effects of cannabis and other drugs [7–9]. Brain maturation during adolescence coincides temporally with key milestones in cognitive development [4, 10]. Anatomical differences between adult and adolescent samples are pronounced in frontal and striatal regions, which are important to reward and motivation [11]. Studies comparing neural reward processing in adults and adolescents suggest that while adolescents recruit a fronto-striatal network largely overlapping with that of adults [12], they may also show hyperactivity in limbic and striatal reward systems [13–15]. There are no studies to date comparing reward processing between adult and adolescent cannabis users.

Anticipation of monetary reward has been linked with increased activity in the ventral striatum, thalamus, anterior insula, premotor cortex, and medial prefrontal cortex, and monetary reward outcome/feedback with the ventral striatum, orbitofrontal/ventromedial prefrontal cortex (vmPFC), amygdala, and posterior cingulate cortex [16, 17]. The ventral striatum is of particular interest due to its theoretical involvement in addiction [18], and previous research has linked long-term recreational drug use and dependence with blunted ventral striatal activity during reward anticipation [19, 20]. Striatal hypoactivity has not typically been found in cross-sectional functional magnetic resonance imaging (fMRI) studies of cannabis users, either among adults [21–24], or adolescents [25, 26]. However, in a longitudinal study of 108 young adults by Martz et al. [27], greater cannabis use predicted attenuated nucleus accumbens (NAc) responses during reward anticipation, while controlling for other substance use and family history of substance use disorder. Thus, cannabis use in young adulthood may be prospectively associated with blunted activity in the NAc/ventral striatum during reward anticipation. Conversely, previous research has not revealed differences between cannabis users and controls in ventral striatal activity during reward feedback [28]. Some studies have shown an association between cannabis use and increased prefrontal and dorsal striatal activity during feedback [21, 29, 30], though this has not been consistently found [28].

The mixed results of previous studies raise several questions. Firstly, with the exception of Martz et al., previous studies have typically included small samples of 15–20 cannabis users. While these are common sample sizes in fMRI research, replication with larger samples is needed to determine whether differences in findings are due to unmeasured confounders, lack of power, or statistical variability around a null effect. Secondly, there have been no reward processing studies comparing adult and adolescent cannabis users directly along with age-matched control groups. Therefore, previous research gives little indication of whether adolescent cannabis users are more likely to show changes in neural reward processing compared to adult cannabis users, as might be expected based on the hypothesised adolescent vulnerability to harmful effects of cannabis.

In the present study, we investigated reward anticipation and reward feedback in 125 adolescent and adult cannabis users and controls during fMRI. We proposed the following, pre-registered [31] hypotheses:

1. Cannabis users will show lower activity in the bilateral ventral striatum during anticipation of reward, compared to controls.
2. There will be a significant interaction between User-Group and Age-Group on ventral striatal activity during reward anticipation, whereby adolescent cannabis users will show greater attenuation than adult users.

We also conducted a whole-brain analysis to determine differences in other regions. Previous studies have not shown a clear pattern of differences between cannabis users and controls during reward feedback; thus, we did not specify explicit hypotheses of group differences for this phase. The current study is, to our knowledge, the largest fMRI study of reward processing in cannabis users to date, and the first to compare adult and adolescent cannabis users together with age-matched controls.

## Methods

### Participants

The current study presents cross-sectional, baseline data from the longitudinal arm of the CannTeen study [32]. Participants were 35 adolescent cannabis users, 35 adult cannabis users, 35 adolescent controls, and 35 adult controls, recruited from the greater London area via school assemblies, posters and flyers, and social media advertisements. Cannabis users used cannabis at least once per week, on average, over the past 3 months. Adult users were excluded if they had used cannabis frequently before the age of 18, with the objective of isolating the impact of adolescent cannabis use. Controls had ≤10 occasions of cannabis use in their lifetime, and no use in the month prior to the baseline behavioural assessment. Adolescents were 16–17 years of age, and adults were 26–29 years of age. Key exclusion criteria were daily use of psychotropic medication, past-month treatment for any mental health condition, and having used any one illicit drug on more than 6 days over the past 3 months. Full inclusion and exclusion criteria are reported in Supplementary Table S1. All participants provided written and informed consent to participate. The study was conducted in line with the Declaration of Helsinki, and was approved by the University College London (UCL) ethics committee (project ID 5929/003).

### Materials

#### Monetary Incentive Delay task

Reward processing was assessed with the Monetary Incentive Delay (MID) task which includes a reward anticipation phase and a reward outcome/feedback phase [33]. At the start of each trial a cue appeared, which signalled whether the participant could win money on that trial (win trials: orange square) or not (neutral trials: blue square). After the cue followed an anticipation phase, after which a target (white circle) appeared, which the participants had to respond to as quickly as possible in order to win. Participants could win 50 pence on win trials, and there were no loss trials. Full details are presented in the Supplementary materials. The MID is the most commonly used task for assessing neural reward processing in humans [16, 34], and among cannabis users [28].

#### Covariates

Covariates in behavioural and ROI analyses were depression, risk-taking, maternal education, and alcohol, tobacco, and other illicit drug use. These were chosen a priori due to their putative interaction with cannabis use, and reward processing [20, 35–37]. Details are presented in the Supplementary materials.

### Data acquisition

A complete account of data collection procedures is presented in the full study protocol [32]. Participants completed an instant saliva drugs test and a breathalyser, and self-reported abstinence, to confirm no recent use of alcohol or cannabis (≥12 h cut-off) or illicit drugs (≥48 h cut-off) at the start of all study sessions. Drug use was assessed with the timeline followback [38]. Questionnaire, demographic, and drug use information were collected during a baseline behavioural session at the UCL Clinical Psychopharmacology Unit.

The MRI session was typically completed within 2 weeks and always completed within 2 months of the baseline behavioural session, and took place at the Invicro MRI research facility, Hammersmith, London. MRI data were collected with a 3.0T Siemens Magnetom Verio. T_2_* images were acquired using a multiband gradient echo Echo-Planar Imaging sequence [39]. T_1_-weighted structural images were acquired using a Magnetization Prepared Rapid Gradient Echo sequence [40]. Full MRI acquisition parameters are in the Supplementary materials.

### Analyses

Analyses and hypotheses were pre-registered to the Open Science Framework [31]. Behavioural and ROI analyses were performed in R 3.6.2 [41], using the rstatix package [42].

#### Behavioural analyses

Behavioural outcomes on the MID task were success rates (% hit targets) and mean reaction times (RTs) for win and neutral trials. The data were first inspected to ensure that the assumptions of parametric statistics were met. Hit rate and RT were dependent variables in separate fully factorial 2 × 2 × 2 mixed measures analyses of covariance (ANCOVAs), with between-group factors User-Group (control vs. user) and Age-Group (adult vs. adolescent), and within-group factor Trial-Type (win vs. neutral). Covariates were included as specified in the ‘Covariates’ section.

#### Pre-processing and first-level analyses

Pre-processing and first- and second-level fMRI analyses were performed in FSL [43], with the fMRI Expert Analysis Tool [44, 45]. Structural high-resolution images were pre-processed using the fsl_anat script provided with FSL. Functional images were realigned with MCFLIRT (motion correction FMRIB linear image registration tool) [46], and normalised to MNI-152 (Montreal Neurological Institute) space with FNIRT (FMRIB’s nonlinear registration tool), using a 10 mm warp resolution and 12 degrees of freedom. Spatial smoothing was carried out using a 6 mm full-width at half-maximum Gaussian kernel. Raw functional image series, movement estimates, and registration were inspected for each participant.

There were six explanatory variables (EVs): anticipation of win outcomes (Anticipate-win; EV1), anticipation of neutral outcomes (Anticipate-neutral; EV2), feedback on successful win trials (Feedback-win-hit; EV3), feedback on unsuccessful win trials (Feedback-win-miss; EV4), feedback on successful neutral trials (Feedback-neutral-hit; EV5), and feedback on unsuccessful neutral trials (Feedback-neutral-miss; EV6). These were implemented in a general linear model, by convolving their respective onsets with a gamma function model of the hemodynamic response. Motion parameters (standard + temporal derivatives + squared + quadratic) and temporal derivatives were included as regressors-of-no-interest. The FILM pre-whitening procedure was used to account for temporal autocorrelation, and a high-pass filter (100 s cut-off) was used to remove low-frequency noise. Reward anticipation was examined with the Anticipate-win > Anticipate-neutral contrast [1 -1 0 0 0 0], and reward feedback with the Feedback-win-hit > Feedback-win-miss contrast [0 0 1 -1 0 0].

#### Second-level analyses

Second-level analyses were performed with FMRIBs local analysis of mixed effects. Mean blood-oxygen-level-dependent responses across groups were first examined in a whole-brain one-sample *t*-test for the reward anticipation and reward feedback contrasts. We then investigated the main effects of User-Group and Age-Group, and the User-Group × Age-Group interaction with whole-brain *F*-tests for each contrast. Cluster-level statistics were used, with a cluster-defining threshold of *Z* = 3.1 (*p* = 0.001) and a multiple test corrected cluster-extent threshold of *a* = 0.05.

ROI analyses were performed in the bilateral ventral striatum for reward anticipation, and the right ventral striatum and left vmPFC for reward feedback. ROIs were selected based on a meta-analysis by Oldham et al. [16], and defined by constructing 6 mm radius spheres around the coordinates with peak activation likelihood estimates for each contrast (Supplementary Fig. S2). Unstandardised *b*-values were extracted from the lower-level contrasts, and served as the dependent variable in separate fully factorial 2 × 2 ANCOVAs, with factors User-Group and Age-Group. All data were inspected to ensure that the assumptions of parametric statistics were met. Covariates were included as specified in the ‘Covariates’ section. Additional one-sample *t*-tests were performed for all ROIs to assess overall activation across participants, and exploratory bivariate correlations were computed between ROIs and additional cannabis use variables. Finally, Bayesian analyses were performed with values from independent-samples *t*-tests of users compared to controls and adult users compared to adolescent users, using the BayesianFactor package in R [47]. A scaled-information prior of *r* = 0.707 was used, and Jeffreys-Zellner-Siow Bayes factors (BF) above 3 were interpreted as meaningful [48].

## Results

### Sample characteristics

Six participants were excluded due to abnormal behavioural data, as specified in the analysis protocol [31], indicating they had not performed the task correctly. An additional nine participants were excluded due to large head movement or other MRI artefacts. Thus, *n* = 125 participants were carried forward for analysis (see Table 1). Two participants (one adolescent user, one adult control) did not have data on maternal education, and were excluded from the behavioural and ROI analyses. They were not excluded from the whole-brain analyses, as these did not include this covariate.

**Table 1:** Sample characteristics.

	Adolescent users (*n* = 32)	Adult users (*n* = 31)	Adolescent controls (*n* = 31)	Adult controls (*n* = 31)	Group differences
*Demographics and covariates*					
Gender					
Female	16 (50.0%)	14 (45.2%)	15 (48.4%)	16 (51.6%)	ns
Male	16 (50.0%)	17 (54.8%)	16 (51.6%)	15 (48.4%)	
Age in years	17.22 (0.52), 16.31–17.98	27.81 (1.49), 26.27–30.02	17.15 (0.45), 16.27–18.04	27.34 (0.86), 26.10–29.56	Adults > Adolescents***
Ethnicity					
White	23 (71.9%)	24 (77.4%)	19 (61.3%)	22 (71.0%)	
Mixed	6 (18.8%)	2 (6.5%)	5 (16.1%)	0 (0.0%)	
Asian	0 (0.0%)	2 (6.5%)	4 (12.9%)	6 (19.4%)	
Black	2 (6.3%)	2 (6.5%)	2 (6.5%)	2 (6.5%)	
Other	1 (3.1%)	1 (3.2%)	0 (0.0%)	1 (3.2%)	
Prefer not to say	0 (0.0%)	0 (0.0%)	1 (3.2%)	0 (0.0%)	
Maternal education					
Below undergraduate degree	14 (45.2%)	13 (41.9%)	12 (38.7%)	19 (63.3%)	ns
Undergraduate degree or above	17 (54.8%)^a^	18 (58.1%)	19 (61.3%)	11 (36.7%)^a^	
BDI	10.09 (5.88), 1–31	7.71 (10.42), 0–46	9.13 (5.66), 0–26	7.55 (8.90), 0–39	ns
RT-18	11.50 (3.45), 3–18	7.90 (4.04), 3–15	8.52 (4.08), 0–17	7.74 (4.54), 0–16	Users > Controls* Adolescents > Adults**
Alcohol use, days/week	0.87 (0.79), 0–3.25	1.49 (1.31), 0–5.25	0.71 (0.81), 0–3.67	1.49 (1.27), 0–5.25	Adults > Adolescents***
Typical number of units on a day of drinking	7.85 (5.82), 0–29	5.97 (4.78), 0–21	3.63 (4.18), 0–16	3.83 (2.96), 0–15	Users > Controls***
Cigarette/roll-up use, days/week	2.31 (2.82), 0–7	1.45 (2.66), 0–7	0.52 (1.56), 0–6.5	0.55 (1.76), 0–7	Users > Controls**
Cigarettes per day if daily smoker	4.80 (3.27), 1–10, *n* = 5	5.70 (3.11), 2–10, *n* = 5	1 (NA), *n* = 1	13.75 (8.34), 7.5–20, *n* = 2	
Other illicit drug use, monthly use					Users > Controls***
Yes	18 (56.25%)	7 (22.6%)	1 (3.2%)	0 (0.0%)	Adolescents > Adults*
No	14 (43.75%)	24 (77.4%)	30 (96.8%)	31 (100.0%)	
*Cannabis use*					
Ever use (controls)			27 (87.1%)	30 (96.8%)	ns
Number of lifetime uses (controls)			3.61 (3.04), 0–10	4.84 (3.26), 0–10	ns
Days/week of use (users)	3.23 (2.16), 0.83–6.92	3.82 (2.14), 0.75–6.92			ns
Grams used on a day of use (users)	0.90 (0.76), 0.15–4	0.77 (0.88), 0.03–3.5^a^			ns
Hours since last use (users)^b^	44.39 (32.72), 12.50–136.0	41.58 (45.13), 12.08–185.0			ns
Age of first-ever use (users)	14.65 (1.10), 12.0–16.50	17.64 (3.13), 13.0–24.08			Adults > Adolescents***
Age of first weekly use (users)	15.75 (1.08), 13.25–17.67	22.06 (2.88), 17.0–27.67			Adults > Adolescents***
CUDIT (users)	14.56 (5.58), 5–26	11.87 (5.93), 3–26			ns
DSM-5 severe CUD (users)	13 (40.6%)	6 (19.4%)			ns

For continuous data mean (SD) and range are shown. For categorical data, *n* (%) is shown. One alcohol unit equals 10 ml or 8 g of pure alcohol. Eleven participants had not used alcohol in the past three months. Group differences were investigated with 2 × 2 analyses of variance, independent-samples *t*-tests, or *χ*^2^ tests of independence. Age and hours since last use were assessed at the baseline imaging session. All other variables were assessed at the baseline behavioural session.

*BDI* Beck Depression Inventory, *CUD* cannabis use disorder, *CUDIT* Cannabis Use Disorder Identification Test, *DSM* Diagnostic and Statistical Manual of Mental Disorders, *RT-18* Risk-taking 18.

*p < 0.05.

**p < 0.01.

***p < 0.001.

^a^1 participant missing.

^b^3 adolescent users and 4 adult users had not used cannabis the week before scanning, and therefore had missing values for this variable.

### Behavioural analyses

Descriptive statistics and full results of the behavioural analyses are presented in Supplementary Tables S3 and S4. The main effect for Trial-Type was significant for both success rate (*F*_1,113_ = 17.43, *p* < 0.001, *η*_*p*_^2^ = 0.13) and mean RT (*F*_1,113_ = 4.74, *p* = 0.03, *η*_*p*_^2^ = 0.04). Participants had higher success rates and shorter RTs for win trials compared to neutral trials (mean difference 18.87% and 6 ms). There were no significant main or interaction effects of User-Group or Age-Group.

### Whole-brain analyses

Significant clusters and local maxima are presented in Table 2. Regions were labelled using the Harvard–Oxford cortical and subcortical structural atlases [49–51].

**Table 2:** Whole-brain analysis results for the Monetary Incentive Delay task.

	X	Y	Z	*K*	*Z*
*Reward anticipation across the full sample (anticipate-reward* *>* *anticipate-neutral)*					
Paracingulate gyrus R	4	16	40	114,852	11.1
Local maxima					
Insular cortex R	34	22	−4		10.7
Frontal operculum R	34	22	10		10.7
Anterior cingulate gyrus L	−4	6	42		10.7
Anterior cingulate gyrus R	2	4	40		10.5
*Reward feedback across the full sample (feedback-win-hit* *>* *feedback-win-miss)*					
Occipital pole L	−20	−92	0	72,686	10.1
Local maxima					
Lateral occipital cortex L	−34	−90	2		9.93
Nucleus accumbens R	12	4	−10		9.3
Nucleus accumbens L	−10	6	−10		9.29
Frontal pole R	24	40	54	311	5.51
*Main effect of User-Group for reward feedback (feedback-win-hit* *>* *feedback-win-miss), Users* *>* *Controls*					
Angular gyrus R	44	−54	34	471	4.25
Local maxima					
Supramarginal gyrus (posterior) R	58	−42	24		3.86
Angular gyrus L	−44	−52	38	251	4.28
Local maxima					
Supramarginal gyrus (posterior) L	−56	−52	38		3.96
Frontal pole R	26	66	14	275	4.34
*Main effect of Age-Group for reward feedback (feedback-win-hit* *>* *feedback-win-miss), Adolescents* *<* *Adults*					
Superior frontal gyrus	0	12	66	192	4.13
Local maxima					
Supplementary motor cortex L	−6	8	60		4.02
Superior frontal gyrus R	12	12	74		3.42

X, Y, and Z are coordinates in MNI-space. *K* refers to the number of voxels in the cluster. Peak *Z* values are reported for each cluster, and local maxima within clusters, where relevant.

*L* left, *R* right.

**Fig. 1:**
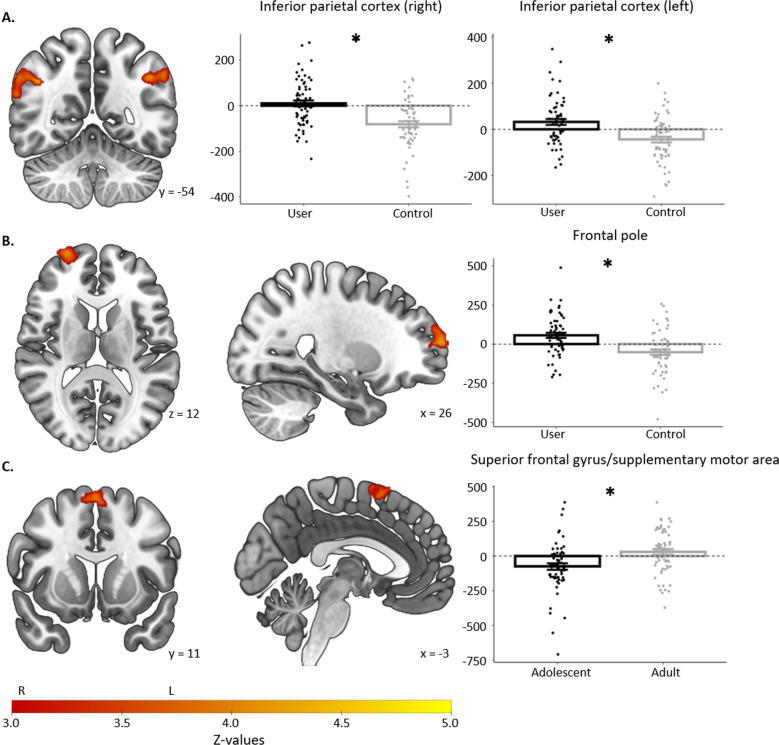
Significant group differences during reward feedback. Regions with significant User-Group or Age-Group differences during reward feedback in the whole-brain analysis, in *n* = 125 participants. Images are presented in radiological orientation, such that left on the image is the right hemisphere. Graphs display means and standard errors for reward feedback beta-values averaged across the given region, with individual values overlayed. **A** User-Group effect in right and left inferior parietal cortex. **B** User-Group effect in the right frontopolar cortex. **C** Age-Group effect in superior frontal gyrus. One-sample *t*-tests showed that individual group means were significantly different from zero, except for in cannabis users in the right inferior parietal cortex, and in adults in the superior frontal gyrus.

The one-sample *t-*test revealed a large cluster of activation during reward anticipation, with peaks in the anterior cingulate cortex and anterior insula (Supplementary Fig. S5). There were no significant main or interaction effects of User-Group or Age-Group in any region during reward anticipation. Peak activation across all participants during reward feedback was found in the occipital cortex, NAc, and frontal pole (Supplementary Fig. S5). There was a significant effect of User-Group in the bilateral inferior parietal cortex, and right frontal pole, with greater activation in users than controls. There was also a significant effect of Age-Group in the bilateral superior frontal gyrus, with greater activation in adults than adolescents. Figure 1 shows regions with significant User-Group or Age-Group differences in reward feedback activity.

### ROI analyses

Full results of the ROI analyses are presented in Supplementary Table S6 and Supplementary Fig. S7. Given that the covariates were not specified in our pre-registered analysis protocol, results without covariates are presented in Supplementary Table S8. Effects of interest were similar in both sets of models.

A one-sample *t*-test confirmed activation in the bilateral ventral striatum during reward anticipation (*t*_122_ = 5.89, *p* < 0.001, *d* = 0.53). There were no significant effects of User-Group (*F*_1,113_ = 0.02, *p* = 0.90, *η*_*p*_^2^ < 0.001), Age-Group (*F*_1,113_ = 1.15, *p* = 0.29, *η*_*p*_^2^ = 0.01), or the User-Group × Age-Group interaction (*F*_1,113_ = 0.01, *p* = 0.91, *η*_*p*_^2^ < 0.001). Bayesian analyses suggested that the null hypothesis of no difference in ventral striatum activity was four times more likely than the alternative when comparing users and controls (BF = 4.54), and three times more likely when comparing adult and adolescent users (BF = 3.21), assuming equal prior probabilities.

There was significant activity in the right ventral striatum (*t*_122_ = 9.34, *p* < 0.001, *d* = 0.84) and the left vmPFC (*t*_122_ = 8.11, *p* < 0.001, *d* = 0.73) during reward feedback. The main effect of User-Group and the User-Group × Age-Group interaction effect were not significant for right ventral striatum (main effect: *F*_1,113_ = 0.20, *p* = 0.66, *η*_*p*_^2^ = 0.002; interaction: *F*_1,113_ = 0.84, *p* = 0.36, *η*_*p*_^2^ = 0.007) or left vmPFC (main effect: *F*_1,113_ = 0.003, *p* = 0.96, *η*_*p*_^2^ < 0.001; interaction: *F*_1,113_ = 0.22, *p* = 0.64, *η*_*p*_^2^ = 0.002). The effect of Age-Group was significant for left vmPFC (*F*_1,113_ = 4.60, *p* = 0.03, *η*_*p*_^2^ = 0.039), but not right ventral striatum (*F*_1,113_ = 0.67, *p* = 0.41, *η*_*p*_^2^ = 0.006). This effect was driven by adults activating more than adolescents. Bayesian analyses supported the null hypothesis of no difference in right ventral striatal feedback activity between users and controls (BF = 5.08) and adult and adolescent users (BF = 3.27). Anecdotal evidence in favour of the null hypothesis was found for left vmPFC feedback activity comparing users and controls (BF = 2.95) [48].

Bivariate correlations between ROIs and additional cannabis use variables are presented in Supplementary Table S9. There was a significant negative correlation between symptoms of cannabis use disorder and feedback activity in the right ventral striatum (*r* = –0.320, *p* = 0.01). This correlation survived correction for multiple comparisons at *q* = 0.1, but not at *q* = 0.05. All other correlations were non-significant.

## Discussion

In the present study, we found that adult and adolescent cannabis users and controls did not differ in neural activity during reward anticipation, or during feedback in pre-defined ROIs. There was no evidence in favour of adolescent vulnerability. Null findings in the ventral striatum for cannabis users compared to controls, and for adult users compared to adolescent users, were supported by Bayesian analyses. However, cannabis users showed hyperactivity in the inferior parietal cortex and right frontopolar cortex during reward feedback, in the whole-brain analysis.

### Reward anticipation and cannabis use

The lack of significant differences in ventral striatum reward anticipation activity was contrary to our hypotheses, though is largely consistent with previous cross-sectional studies of reward anticipation in cannabis users [28]. Even in the longitudinal study by Martz et al. [27], which found a longitudinal effect of cannabis, there was no cross-sectional association between reward anticipation at baseline and previous cannabis use. One previous study did find attenuated anticipation activity in the NAc in cannabis users compared to controls [30], but this study included only 27 users and controls, and did not control for other drug use or mental health variables.

The present sample had a lower frequency of cannabis use compared to some previous fMRI studies of reward processing in cannabis users [24, 29], though similar to others [23, 52, 53]. It is possible that differences would have emerged with more frequent or problematic use. However, bivariate correlations did not reveal a significant association between ventral striatum reward anticipation activity and cannabis use frequency, quantity, length of abstinence, age of onset, symptoms of dependence, or cannabis use disorder (Supplementary Table S9). Thus, the current results, including the Bayesian analyses, and in combination with previous evidence, suggest that there is no association between cannabis use and altered neural responses to reward anticipation cross-sectionally.

It is possible that previous differences between cannabis users and controls were confounded by concurrent use of tobacco/nicotine [20]. Co-use of cannabis and cigarettes is common, and many users consume cannabis together with tobacco in joints [36, 54]. Previous studies have found an association with blunted striatal activity during reward anticipation more consistently for nicotine than for cannabis [19, 26]. The effects of acute cannabis on striatal reward processing have also been shown to differ depending on comorbid nicotine dependence [55]. In addition, in the current study, there was a significant effect of the cigarette covariate (Supplementary Table S6), in the direction of lower ventral striatum anticipation activity with a higher frequency of cigarette use. This was the only significant effect for the reward anticipation models, and suggests that cigarette use may have an effect independent of cannabis use. Future studies should attempt to demarcate the relative contributions of cannabis and tobacco/nicotine on striatal reward anticipation.

### Reward feedback and cannabis use

Previous studies have not found a consistent association between cannabis use and neural responses to reward feedback [28]. In the current study, there were no differences in ROIs pre-defined based on a large meta-analysis [16]. However, a whole-brain analysis revealed greater activity in the right frontopolar cortex and bilateral inferior parietal cortex in cannabis users. Interestingly, this difference was driven by opposite patterns of activity for successful win-trial feedback compared to unsuccessful win-trial feedback in cannabis users and controls. While users activated the right frontopolar and bilateral inferior parietal cortex after a successful win, controls deactivated the same areas. This pattern has not emerged previously, though two earlier studies did find hyperactivity in different regions of the prefrontal cortex in cannabis users during feedback [29, 30], somewhat consistent with the present findings.

The present study found no differences between cannabis users and controls in regions that are commonly activated during reward feedback [16]. The current results may thus not reflect a reward-specific effect, but rather a general trend towards neural hyperactivation in cannabis users. Several previous studies have found increased fronto-parietal activation in cannabis users during cognitive tasks [56], especially in adolescent users [57]. This has been proposed to reflect a neural compensatory mechanism in cannabis users, such that greater activation helps to achieve normal levels of behavioural performance [58, 59]. Accordingly, hyperactivation in frontopolar and inferior parietal cortex in cannabis users may have occurred in order to achieve similar performance to controls on the MID task in the current study. However, this interpretation is speculative. The present differences in fronto-parietal activity may also reflect pre-existing vulnerabilities in cannabis users relative to controls, such as attentional disparities [60, 61]. Longitudinal studies are needed to establish the potential mechanisms underlying differences in neural reward feedback activity between cannabis users and controls.

### No adolescent vulnerability

Most crucially, the present results did not support our hypotheses of an interaction between User-Group and Age-Group on reward anticipation. There was also no significant interaction for feedback. Results were supported by Bayesian analyses. Adolescent and adult users were well matched in frequency and quantity of use, and group differences were generally in the direction of higher levels among adolescent users on variables that would likely confer greater risk (i.e., abuse and dependence, early onset of use, other drug use). Thus, our results suggest that adolescent cannabis users are not at greater risk of altered neural reward processing compared to adult users. This could be because cannabis does not disrupt the reward system non-acutely, regardless of age. Alternatively, it may be that the striatal reward system has matured by age 16–17 and is therefore not sensitive to disruption at this age [15, 62]. Importantly, there is evidence that adolescent users are at greater risk of other cannabis-related adverse outcomes, such as dependence [63–65]. Moreover, adolescent cannabis users may be more vulnerable to syndromes of disrupted reward processing, such as anhedonia [66]. Therefore, psychological and behavioural differences may still exist in the absence of significant differences in neural activity in pre-defined brain areas, using the present task and methods. A brief discussion of the main age-group effect is provided in the Supplementary materials.

### Clinical and policy implications

Although the present neuroimaging study is not clinical in nature, it has some relevance to treatment strategy. Our findings are inconsistent with models suggesting that cannabis dependence is subserved by blunted neural responsiveness to non-cannabis rewards [67, 68]. Thus, we should not assume anticipatory neural processing of natural rewards is impaired in chronic cannabis users and require treatments to redress a purported deficiency. This is not to say that treatment should ignore natural rewards, as contingency management and behavioural activation may have efficacy in the treatment of cannabis use disorder [69, 70]. Future research should integrate fMRI into clinical trials or observational treatment studies to examine the complex role of neural reward processing in illness and recovery. Our findings are also relevant to the development of evidence-based harm reduction strategies, use of medical cannabis, and drug education, with knowledge of the long-term effects on brain and cognition imperative. Finally, as more countries regulate recreational and medical cannabis, minimum age-of-purchase limits will be implemented. Our results suggest 16–17-year olds are not at greater risk of cannabis-related disruption of neural reward processing. Nevertheless, adolescent vulnerability to other cannabis-related harms should be investigated, and influence policy development.

### Strengths and limitations

The most notable limitation of the current study is the cross-sectional design. This means that we cannot exclude the possibility that pre-existing group differences obscured an effect of cannabis use on reward processing. However, key confounders including depression, risk-taking, maternal education, and alcohol, cigarette, and illicit drug use were controlled statistically in ROI and behavioural analyses, and groups were carefully matched on key variables. Another limitation concerns our use of monetary reward exclusively. It may be that the reward system shows greater changes in response to other rewards, such as cannabis cues, which could also be linked with craving and withdrawal symptoms [71]. Strengths of the current study include the relatively large sample size, pre-registration of analyses, rigorous assessment of cannabis and other drug use with the timeline followback, biological verification of recent abstinence, good control of important confounders, matching of adolescent and adult users for the level of cannabis use, and the novel comparison of adult and adolescent user-groups with age-matched controls. Thus, the current study was well-powered to detect a difference between cannabis users and controls, compared to previous research.

## Conclusion

In the present study, cannabis users were found to overactivate fronto-parietal networks relative to controls during reward feedback. Future longitudinal studies are needed to corroborate this. Future studies should include adolescent users in particular, and continue to adjust for important confounders such as tobacco/nicotine use. Overall, our results suggest that reward anticipation and feedback processing in key reward regions are unaffected by cannabis use at a moderate frequency of 3 to 4 days per week, and that adolescents are not at increased vulnerability to cannabis-related differences in neural reward processing.

## Supplementary information

Supplementary materials
