# Supplementary material for: Neural responses to reward anticipation and feedback in adult and adolescent cannabis users and controls
Source: Neuropsychopharmacology. 2022 Apr 6;47(11):1976–83. doi: 10.1038/s41386-022-01316-2 (PMC9485226; doi:10.1038/s41386-022-01316-2)
Supplement: Supplementary file 1 — Supplementary materials [file 41386_2022_1316_MOESM1_ESM.docx]

**Title:** Neural responses to reward anticipation and feedback in adult and adolescent cannabis users and controls

**SUPPLEMENTARY MATERIALS**

**Authors:** Martine Skumlien MRes^1,2^, Claire Mokrysz PhD^2^, Tom P Freeman PhD^2,3^, Matthew B Wall PhD^2,4,5^, Michael Bloomfield PhD^6^, Rachel Lees MSc^2,3^, Anna Borissova MBBS^2^, Kat Petrilli MRes^2,3^, James Carson MRes^2^, Tiernan Coughlan MSc^2^, Shelan Ofori MSc^2^, Christelle Langley PhD^1,7^, Barbara J Sahakian PhD DSc^1,7^, H Valerie Curran PhD^2^, Will Lawn PhD^2,8^

**Affiliations:**

^1^Department of Psychiatry, University of Cambridge, Cambridge, UK; ^2^Clinical Psychopharmacology Unit, Clinical Educational and Health Psychology Department, University College London, London, UK; ^3^Addiction and Mental Health Group (AIM), Department of Psychology, University of Bath, Bath, UK; ^4^Invicro, London, UK; ^5^Faculty of Medicine, Department of Metabolism, Digestion and Reproduction, Imperial College London, London, UK; ^6^Division of Psychiatry, University College London, London, UK; ^7^Behavioural and Clinical Neuroscience Institute, University of Cambridge, UK; ^8^National Addiction Centre, Institute of Psychiatry Psychology and Neuroscience, King’s College London, London, UK

**Section 1. Supplemental methods**

**Table S1.** Inclusion and exclusion criteria for all participants, and specific criteria for each group.

|  | Inclusion criteria | Exclusion criteria |
| --- | --- | --- |
| All participants | - Able to come to University College London five times over the next year - Capacity to give informed consent - Normal or corrected-to-normal vision - Fluent in English - Right-handed - Able to come to Invicro during normal working hours | - Any illicit drug use within 48 hours of the behavioural baseline session, verified with self-report and saliva testing - Any cannabis or alcohol use within 12 hours of the behavioural baseline session, verified with self-report and saliva/breathalyser testing. - Personal history of a diagnosed psychotic episode or disorder - Any one illicit drug taken >2 days/month (averaged over last 3 months) (except laughing gas) - Use of laughing gas >1 day/week (averaged over last 3 months) - Receiving treatment for any mental health condition, including cannabis dependence, in the last month - Unwilling to give blood samples or likely to faint on blood sampling - Current daily use of a medication which is commonly psychotropic - Any mental or physical health problem judged to be problematic for the study, by a medical doctor - Pregnant - Piercings that can’t be removed - Pacemaker - Hearing aid - Full braces - Any other MRI contraindication as judged by Invicro radiographers |
| Teenage cannabis users | - Aged 16-17 years - Cannabis use at a frequency of 1-7 days/week (averaged over last 3 months) | - Age-adjusted BMI <2^nd^ percentile or >99.6^th^ percentile |
| Teenage controls | - Aged 16-17 years - Between 1 and 10 days of lifetime cannabis use ***or*** 0 days of lifetime cannabis use and at least 1 day of lifetime cigarette/roll-up use | - Age-adjusted BMI <2^nd^ percentile or >99.6^th^ percentile - Cannabis use more than once in the last 3 months before behavioural baseline session - Cannabis use in the month prior to the behavioural baseline session |
| Adult cannabis users | - Aged 26-29 years - Cannabis use at a frequency of 1-7 days/week (averaged over last 3 months) | - Before the age of 18, cannabis use at a frequency of once per week or more for a period of 3 months or more. - BMI <18.5 or BMI>34.9 |
| Adult controls | - Aged 26-29 years - Between 1 and 10 days of lifetime cannabis use ***or*** 0 days of lifetime cannabis use and at least 1 day of lifetime cigarette/roll-up use | - Cannabis use more than once in the last 3 months before behavioural baseline session - Cannabis use in the month prior to the baseline session - BMI <18.5 or BMI>34.9 |

Abbreviations: *BMI* Body Mass Index, *MRI* magnetic resonance imaging.

*Monetary Incentive Delay task.* The current version of the Monetary Incentive Delay (MID) task included win and neutral trials, but no loss trials. At the start of each trial, a cue appeared for 500 ms, which signalled whether the participant could win money on that trial (win trials: orange square) or not (neutral trials: blue square). After the cue disappeared there was an anticipation phase for 2-4 s (jittered, blank screen), after which a white circle appeared, which the participant responded to by pressing a button on a button-box. Participants were instructed to respond to the white circle as quickly as possible, even if they could not win any money on that trial. After a response had been made, the participants received feedback indicating whether they were successful on that trial, and whether they won any money. The required response time for a hit was calibrated to each participant’s performance, to obtain a 50% hit rate. The initial threshold was set at 300 ms, and was reduced by 16.66 ms (one screen refresh) after each ‘hit’, down to a minimum of 250 ms, or increased by 16.66 ms after each ‘miss’, up to a maximum of 400 ms. There were 66 trials in total, of which 38 were neutral trials and 28 were win trials, with a jittered inter-trial interval between 1.2 s and 9.2 s.

### *Covariates.* Depressive symptomatology was assessed with the Beck Depression Inventory (BDI) [1]. Risk-taking was assessed with the risk-taking 18 (RT-18) questionnaire [2]. Maternal education was measured as below undergraduate degree and undergraduate degree or above. All drug use was assessed with the timeline followback [3]. Alcohol and non-cannabis tobacco use were measured as average days per week of use over the past three months. Other illicit drug use was measured as any illicit drug used on average once per month over the past three months, or less than this.

*Additional measures.* Instant saliva drugs tests were either Alere DDSV 703 or ALLTEST DSD-867MET/C, which tested for cocaine, Δ^9^-tetrahydrocannabinol (THC), opiates, amphetamine, methamphetamine, and benzodiazepines. A Lion Alcometer 500 breathalyser was used to measure blood alcohol concentration (BAC). Participants with a BAC >0 or positive result for any illicit drug (including THC) were rescheduled.

*MRI data acquisition and analysis.* Participants practised the MID task before going into the scanner, and then performed the task while in the scanner alongside other tasks, which will be reported elsewhere. MRI data were collected with a 3.0 T Siemens Magnetom Verio, using a 32-channel phased array head coil. T_2_* images were acquired using a multiband gradient echo Echo-Planar Imaging (EPI) sequence (repetition time, TR=1250 ms, echo time, TE=30 ms, flip angle=62°) [4]. A total of 484 volumes were collected for each participant, with a field-of-view of 192 mm and a matrix size of 64x64 mm, yielding an in-plane resolution of 3x3 mm. Slice thickness was 3 mm, resulting in 3 mm isotropic voxels. Forty-four slices were collected using interleaved acquisition, and a multi-band acceleration factor of 2. T_1_-weighted structural images were acquired using a Magnetization Prepared Rapid Gradient Echo (MPRAGE) sequence (TR=2300 ms, TE=2.98 ms, flip angle=9°, parallel imaging acceleration factor=2) [5], with a spatial resolution of 1 mm isotropic voxels.

For MID task reward feedback, previous studies have used two variants: Feedback-win-hit > Feedback-neutral-hit [0 0 1 0 -1 0], and Feedback-win-hit > Feedback-win-miss [0 0 1 -1 0 0]. Given that the Feedback-win-hit > Feedback-win-miss contrast has been shown to be more sensitive to task-related differences in activity [6], this was the main reward feedback contrast in the current study. However, we also examined the Feedback-win-hit > Feedback-neutral-hit contrast for comparison.


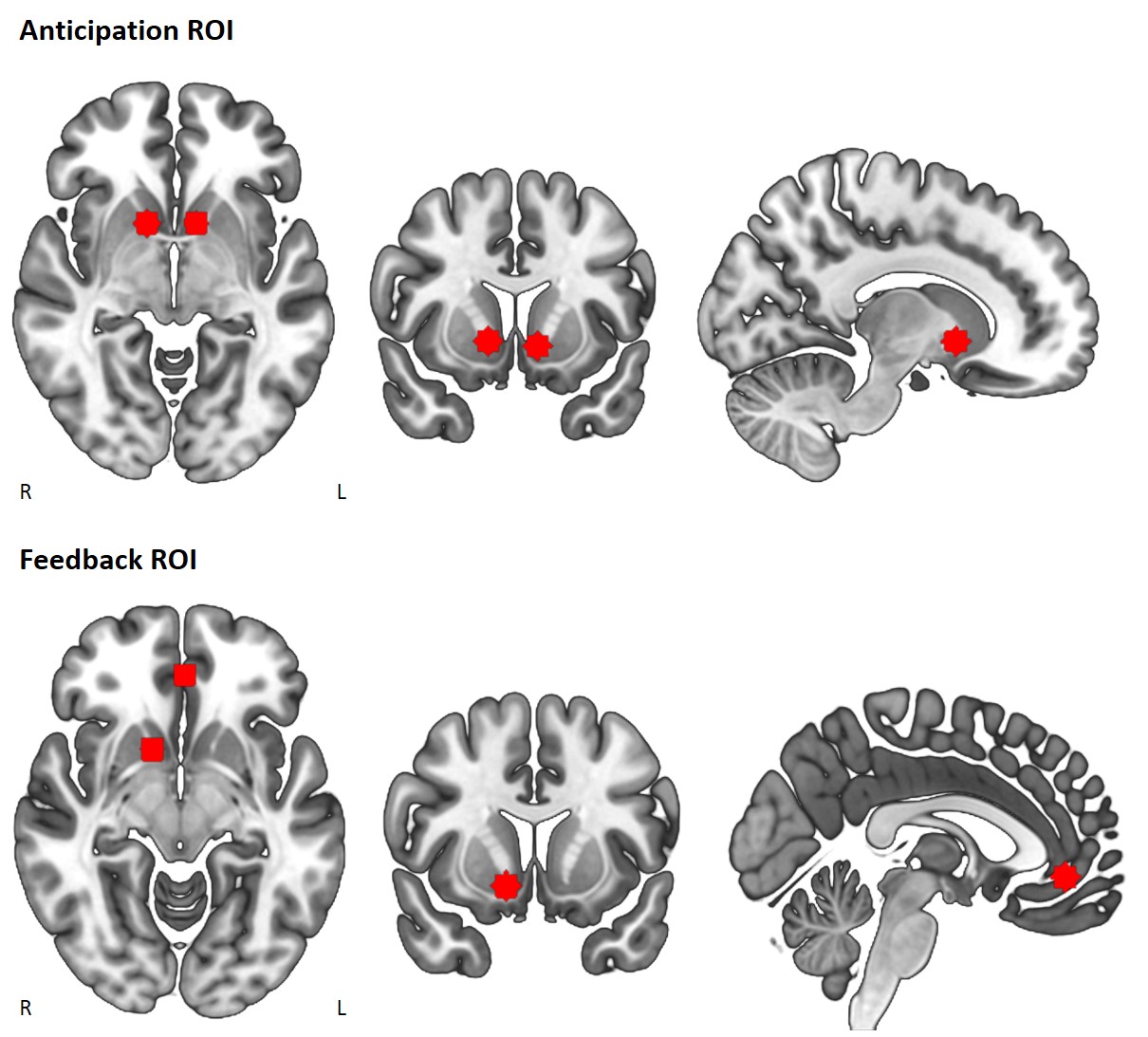


**Fig. S2 Regions of interest (ROIs) for reward anticipation and feedback.** ROIs were based on the results of Oldham et al. [6]. Centre of sphere was x = 12, y = 10, z = -4 for the right ventral striatum and x = -10, y = 10, z = -6 for the left ventral striatum, combined into a single ROI, for reward anticipation. Centre of sphere was x = 12, y = 10, z = -10 for the right ventral striatum and x = -2, y = 42, z = -6 for left ventromedial prefrontal cortex for reward feedback. Images are presented in radiological orientation, such that left on the image is the right hemisphere.

**Section 2. Supplemental results**

**Table S3.** MID behavioural descriptive statistics

|  | **Adolescent users**  (*n* = 31) | **Adolescent controls**  (*n* = 31) | **Adult users**  (*n* = 31) | **Adult controls**  (*n* = 30) |
| --- | --- | --- | --- | --- |
| Mean (SD) |  |  |  |  |
| Win % hit | 61.06 (8.00) | 60.25 (8.08) | 59.22 (7.25) | 63.10 (7.46) |
| Neutral % hit | 42.53 (6.23) | 42.36 (6.20) | 43.72 (6.58) | 39.39 (6.43) |
| Win RT (ms) | 237 (21) | 239 (23) | 235 (20) | 241 (20) |
| Neutral RT (ms) | 240 (21) | 245 (25) | 242 (22) | 250 (20) |

*MID* Monetary Incentive Delay task, *ms* milliseconds, *RT* response time, *SD* standard deviation.

**Table S4.** MID behavioural analyses full results

|  | **F** | **df** | **p** | **η_p_^2^** |
| --- | --- | --- | --- | --- |
| **% Hit** |  |  |  |  |
| Trial-Type | 17.43 | 1, 113 | <.001 | .134 |
| User-Group | 2.00 | 1, 113 | .16 | .017 |
| Age-Group | 1.20 | 1, 113 | .28 | .010 |
| Trial-Type*User-Group | 0.91 | 1, 113 | .34 | .008 |
| Trial-Type*Age-Group | 0.19 | 1, 113 | .66 | .002 |
| User-Group*Age-Group | 0.27 | 1, 113 | .61 | .002 |
| Trial-Type*User-Group*Age-Group | 3.85 | 1, 113 | .052 | .033 |
| BDI | 2.66 | 1, 113 | .11 | .023 |
| RT-18 | <.001 | 1, 113 | .99 | <.001 |
| Alcohol | 2.49 | 1, 113 | .12 | .022 |
| Cigarettes/roll-ups | 0.21 | 1, 113 | .64 | .002 |
| Illicit drugs | 1.47 | 1, 113 | .23 | .013 |
| Maternal education | 0.18 | 1, 113 | .68 | .002 |
| Trial-Type*BDI | 2.85 | 1, 113 | .09 | .025 |
| Trial-Type*RT-18 | 0.002 | 1, 113 | .96 | <.001 |
| Trial-Type*Alcohol | 0.17 | 1, 113 | .68 | .002 |
| Trial-Type*Cigarettes/roll-ups | 0.02 | 1, 113 | .88 | <.001 |
| Trial-Type*Illicit drugs | 0.93 | 1, 113 | .34 | .008 |
| Trial-Type*Maternal education | 0.19 | 1, 113 | .66 | .002 |
| **RT** |  |  |  |  |
| Trial-Type | 4.74 | 1, 113 | .03 | .040 |
| User-Group | 0.03 | 1, 113 | .86 | <.001 |
| Age-Group | 0.03 | 1, 113 | .85 | <.001 |
| Trial-Type*User-Group | 0.62 | 1, 113 | .43 | .005 |
| Trial-Type*Age-Group | 2.89 | 1, 113 | .09 | .025 |
| User-Group*Age-Group | 0.67 | 1, 113 | .42 | .006 |
| Trial-Type*User-Group*Age-Group | 0.24 | 1, 113 | .62 | .002 |
| BDI | 0.03 | 1, 113 | .86 | <.001 |
| RT-18 | 1.29 | 1, 113 | .26 | .011 |
| Alcohol | 0.03 | 1, 113 | .86 | <.001 |
| Cigarettes/roll-ups | 0.02 | 1, 113 | .88 | <.001 |
| Illicit drugs | 1.62 | 1, 113 | .21 | .014 |
| Maternal education | 0.13 | 1, 113 | .72 | .001 |
| Trial-Type*BDI | 3.11 | 1, 113 | .08 | .027 |
| Trial-Type*RT-18 | 0.45 | 1, 113 | .50 | .004 |
| Trial-Type*Alcohol | 1.15 | 1, 113 | .29 | .010 |
| Trial-Type*Cigarettes/roll-ups | 0.60 | 1, 113 | .44 | .005 |
| Trial-Type*Illicit drugs | 0.72 | 1, 113 | .40 | .006 |
| Trial-Type*Maternal education | 0.38 | 1, 113 | .54 | .003 |

*BDI* Beck Depression Inventory, *MID* Monetary Incentive Delay task, *RT-18* risk-taking 18.


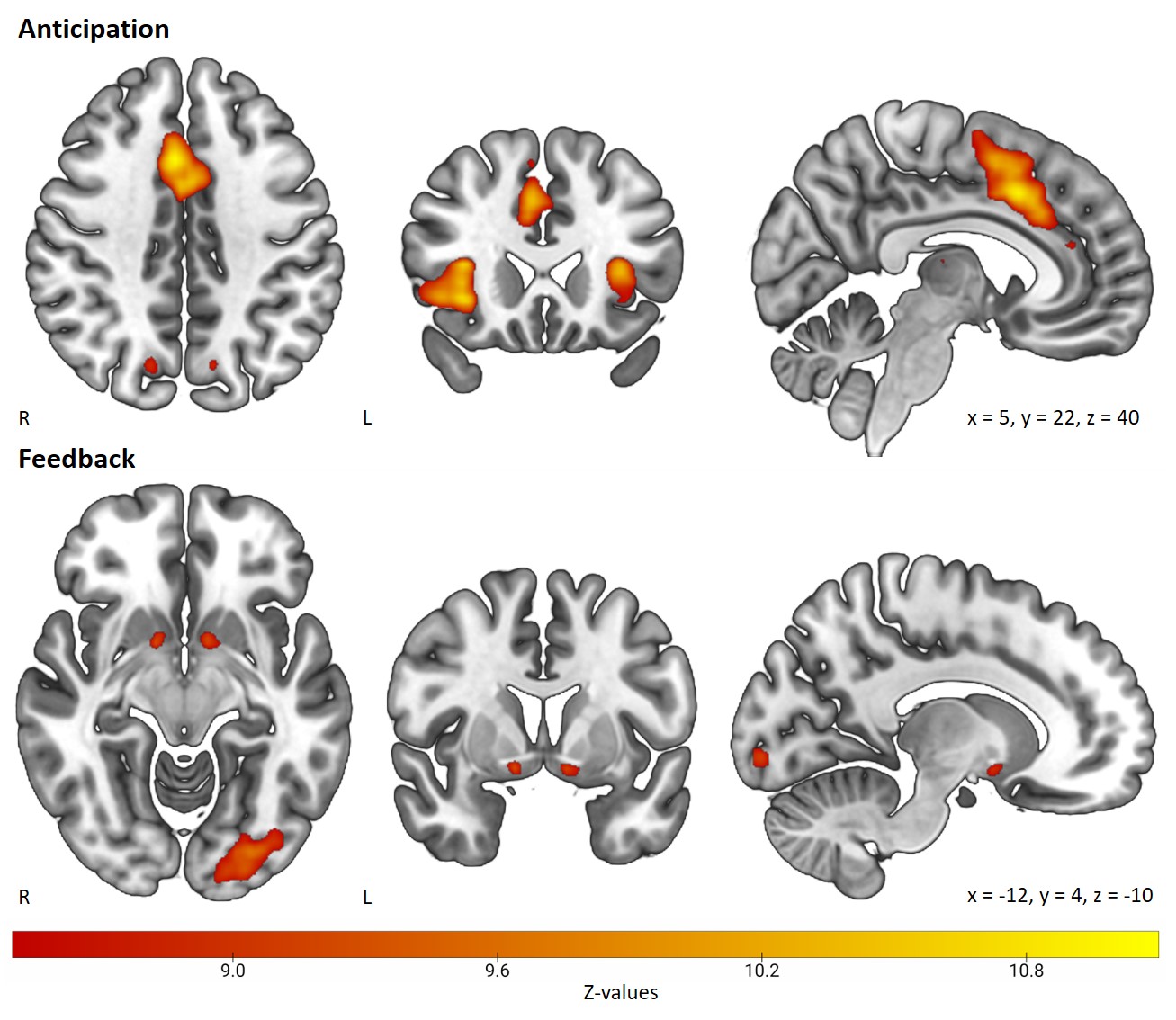


**Fig. S5 Reward anticipation and feedback activity for the full sample.** Significant activation for reward anticipation (top) and reward feedback (bottom) across the full sample of *n*=125 participants. Images are presented in radiological orientation, such that left on the image is the right hemisphere. A higher threshold of Z=8.5 was used for visualisation purposes.

*Secondary feedback contrast.* There were no significant effects of User-Group, Age-Group, or the User-Group*Age-Group interaction for the secondary Feedback-win-hit > Feedback-neutral-hit contrast.

**Table S6.** ROI analyses full results

|  | **F** | **df** | **p** | **η_p_^2^** |
| --- | --- | --- | --- | --- |
| **Reward anticipation, bilateral ventral striatum** |  |  |  |  |
| User-Group | 0.02 | 1, 113 | .90 | <.001 |
| Age-Group | 1.15 | 1, 113 | .29 | .010 |
| User-Group*Age-Group | 0.01 | 1, 113 | .91 | <.001 |
| BDI | 0.29 | 1, 113 | .59 | .003 |
| RT-18 | 0.001 | 1, 113 | .98 | <.001 |
| Alcohol | 0.85 | 1, 113 | .36 | .007 |
| Cigarettes/roll-ups | 10.49 | 1, 113 | .002 | .085 |
| Illicit drugs | 0.37 | 1, 113 | .54 | .003 |
| Maternal education | 2.61 | 1, 113 | .11 | .023 |
| **Reward feedback, right ventral striatum** |  |  |  |  |
| User-Group | 0.20 | 1, 113 | .66 | .002 |
| Age-Group | 0.67 | 1, 113 | .41 | .006 |
| User-Group*Age-Group | 0.84 | 1, 113 | .36 | .007 |
| BDI | 1.72 | 1, 113 | .19 | .015 |
| RT-18 | 2.42 | 1, 113 | .12 | .021 |
| Alcohol | 0.87 | 1, 113 | .35 | .008 |
| Cigarettes/roll-ups | 0.50 | 1, 113 | .48 | .004 |
| Illicit drugs | 0.15 | 1, 113 | .70 | .001 |
| Maternal education | 1.94 | 1, 113 | .17 | .017 |
| **Reward feedback, left ventromedial prefrontal cortex** |  |  |  |  |
| User-Group | 0.003 | 1, 113 | .96 | <.001 |
| Age-Group | 4.60 | 1, 113 | .03 | .039 |
| User-Group*Age-Group | 0.22 | 1, 113 | .64 | .002 |
| BDI | 2.71 | 1, 113 | .10 | .023 |
| RT-18 | 0.09 | 1, 113 | .77 | .001 |
| Alcohol | 0.97 | 1, 113 | .33 | .008 |
| Cigarettes/roll-ups | 0.41 | 1, 113 | .52 | .004 |
| Illicit drugs | 1.70 | 1, 113 | .19 | .015 |
| Maternal education | 0.004 | 1, 113 | .95 | <.001 |

Abbreviations as in Table S3.


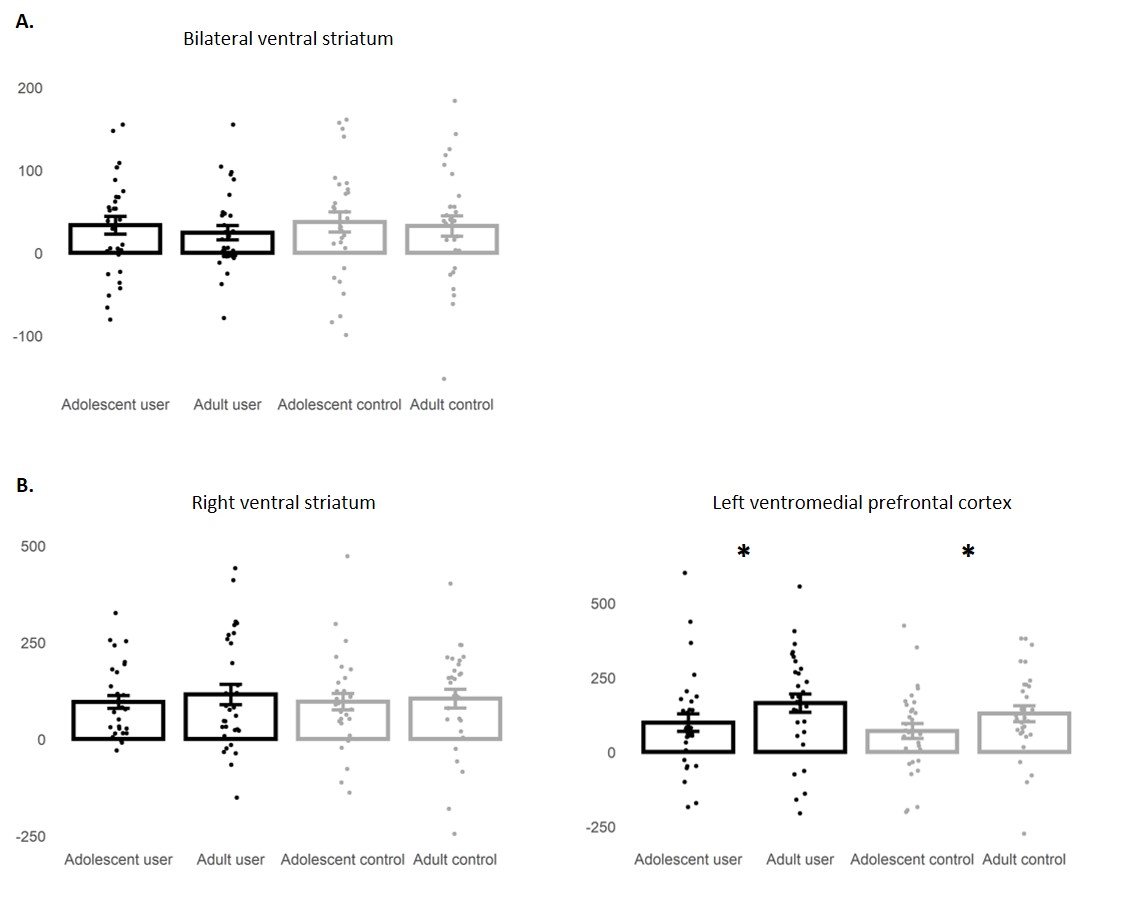


**Fig. S7 Region of interest (ROI) activity by group.** ROI means and standard errors by group, with individual values overlayed. **A** Reward anticipation. **B** Reward feedback.

**Table S8.** ROI analyses full results without covariates

|  | **F** | **df** | **p** | **η_p_^2^** |
| --- | --- | --- | --- | --- |
| **Reward anticipation, bilateral ventral striatum** |  |  |  |  |
| User-Group | 0.28 | 1, 121 | .60 | .002 |
| Age-Group | 0.44 | 1, 121 | .51 | .004 |
| User-Group*Age-Group | 0.08 | 1, 121 | .78 | .001 |
| **Reward feedback, right ventral striatum** |  |  |  |  |
| User-Group | 0.06 | 1, 121 | .82 | ≤ .001 |
| Age-Group | 0.37 | 1, 121 | .54 | .003 |
| User-Group*Age-Group | 0.17 | 1, 121 | .68 | .001 |
| **Reward feedback, left ventromedial prefrontal cortex** |  |  |  |  |
| User-Group | 1.56 | 1, 121 | .21 | .013 |
| Age-Group | 4.41 | 1, 121 | .04 | .035 |
| User-Group*Age-Group | 0.04 | 1, 121 | .83 | ≤ .001 |

*Note*. These analyses included the two subjects who were excluded from the main analyses due to missing maternal education.

**Table S9**. Bivariate correlations between regions of interest and cannabis use variables, within cannabis users (*n*=63).

|  | **Anticipation, bilateral ventral striatum** | **Feedback, right ventral striatum** | **Feedback, left ventromedial prefrontal cortex** |
| --- | --- | --- | --- |
| Days/week of cannabis use (*n*=63) | -.220 | -.147 | .013 |
| Grams used on a day of use (*n*=62) | -.222 | -.106 | -.074 |
| Hours since last use (*n*=56) | .205 | .053 | .077 |
| Age of first ever use (*n*=63) | .078 | .083 | -.015 |
| Age of first weekly use (*n*=63) | -.080 | .073 | .054 |
| CUDIT (*n*=63) | .001 | -.132 | -.119 |
| DSM-5 CUD number of symptoms (*n*=63) | .063 | -.320* | -.145 |

**p*<.05

*CUD* Cannabis Use Disorder, *CUDIT* Cannabis Use Disorder Identification Test, *DSM* Diagnostic and Statistical Manual of Mental Disorders.

*Note.* The significant correlation between DSM-5 CUD number of symptoms and feedback activity in the right ventral striatum survived multiple comparisons correction with the Benjamin-Hochberg false discovery rate (FDR) procedure at FDR of 10%, but not at FDR of 5%.

**Section 3. Supplemental discussion**

In addition to our two main hypotheses, we also hypothesised that adolescents would show elevated activity in the ventral striatum during anticipation of reward, compared to adults [7]. Our results were not consistent with this hypothesis. In fact, adults were found to overactivate prefrontal and supplementary motor areas, relative to adolescents (see Table 2, Table S6, Fig. S7). Though some previous studies have found the opposite pattern of neural hyperactivity in the adolescent reward system, findings are mixed, and may be dependent on task parameters [8]. Given the present focus on cannabis use, our sample was not intended to be representative of UK adults and adolescents, which may have influenced our findings. Larger scale studies which specifically aim to investigate reward processing in adults and adolescents will yield more reliable results.

**References**

1. Beck AT, Steer RA, and Brown GK, Beck depression inventory-II. The Psychological Corporation: San Antonio, Texas; 1996.

2. de Haan L, Kuipers E, Kuerten Y, van Laar M, Olivier B, and Verster JC. The RT-18: a new screening tool to assess young adult risk-taking behavior*.* Int J Gen Med. 2011;4:575-84.

3. Robinson SM, Sobell LC, Sobell MB, and Leo GI. Reliability of the Timeline Followback for cocaine, cannabis, and cigarette use*.* Psychol Addict Behav. 2014;28:154-62.

4. Demetriou L, Kowalczyk OS, Tyson G, Bello T, Newbould RD, and Wall MB. A comprehensive evaluation of increasing temporal resolution with multiband-accelerated protocols and effects on statistical outcome measures in fMRI*.* Neuroimage. 2018;176:404-416.

5. Jack CR, Jr., Bernstein MA, Fox NC, Thompson P, Alexander G, Harvey D, et al. The Alzheimer's Disease Neuroimaging Initiative (ADNI): MRI methods*.* J Magn Reson Imaging. 2008;27:685-91.

6. Oldham S, Murawski C, Fornito A, Youssef G, Yucel M, and Lorenzetti V. The anticipation and outcome phases of reward and loss processing: A neuroimaging meta-analysis of the monetary incentive delay task*.* Hum Brain Mapp. 2018;39:3398-3418.

7. Skumlien M, Mokrysz C, Wall MB, Freeman TP, Langley C, Sahakian BJ, et al. Neural response to reward anticipation and feedback in adult and adolescent cannabis users. 2020. <https://osf.io/aehc9/>. Accessed 22 September 2021.

8. Richards JM, Plate RC, and Ernst M. A systematic review of fMRI reward paradigms used in studies of adolescents vs. adults: the impact of task design and implications for understanding neurodevelopment*.* Neurosci Biobehav Rev. 2013;37:976-91.
